# Elucidating molecular mechanisms and therapeutic synergy: irreversible HER2-TKI plus T-Dxd for enhanced anti-HER2 treatment of gastric cancer

**DOI:** 10.1007/s10120-024-01478-6

**Published:** 2024-02-22

**Authors:** Jiankun Liu, Tienian Zhu, Ruijing Zhao, Wenjun Ren, Fei Zhao, Jingpu Liu

**Affiliations:** 1 Department of Oncology, Hebei Medical University, Shijiazhuang, 050017 Hebei China; 2 Department of Medical Oncology, Bethune International Peace Hospital, Shijiazhuang, 050082 Hebei China; 3 Department of Immunology, Hebei Medical University, Key Laboratory of Immune Mechanism and Intervention On Serious Disease in Hebei Province, Shijiazhuang, 050017 Hebei China

**Keywords:** ErbB-2 receptor (HER2), Gastric cancer (GC), Endocytoses, Ubiquitylation

## Abstract

**Background:**

HER2-targeted therapies have improved the outcomes of HER2-positive gastric cancer (GC), yet resistance remains a challenge. We sought to explore the effects of reversible and irreversible HER2 tyrosine kinase inhibitors (TKIs) alone or in combination with the HER2-targeting antibody drug conjugate trastuzumab deruxtecan (T-Dxd).

**Methods:**

The effects of HER2-TKIs on HER2 and downstream signaling were evaluated via Western blotting. Proteasomal inhibitors and co-immunoprecipitation assays were performed to explore the role of proteasomal degradation in HER2 expression modulation, and immunofluorescence assays were employed to explore mechanisms of HER2 internalization. The synergistic potential of the irreversible HER2-TKI pyrotinib in combination with T-Dxd was validated using growth and viability assays in anti-HER2-positive GC cell cultures and tumor growth and immunohistochemical staining assays in a mouse xenograft model.

**Results:**

Our study revealed that reversible HER2-TKIs elevated HER2 protein levels, whereas irreversible HER2-TKIs decreased them. Pyrotinib triggered HER2 degradation within the proteasome by promoting ubiquitination and dissociation from HSP90. Furthermore, pyrotinib substantially induced HER2 internalization, which led to improved cellular uptake of T-Dxd. The increased T-Dxd uptake was accompanied by greater efficacy in suppressing the growth of GC cells and enhanced anti-tumor effects in an animal model.

**Conclusion:**

In summary, our research reveals the molecular mechanisms of irreversible HER2-TKIs in regulating HER2 protein expression by promoting HER2 internalization. These findings advance our comprehension of targeted therapy for GC and provide a promising therapeutic combination strategy with enhanced efficacy against HER2-positive GC.

**Supplementary Information:**

The online version contains supplementary material available at 10.1007/s10120-024-01478-6.

## Introduction

Gastric cancer (GC) is a prevalent malignancy within the gastrointestinal tract, with China alone contributing to approximately 50% of the global incidence and mortality [1]. HER2-positive GC, characterized by its high malignancy and dismal prognosis, yields a 5-year survival rate of only 5–20% [2]. HER2 overexpression occurs in roughly 7.3–20.2% of advanced GC [3]; and as a result, HER2 targeting has become pivotal in HER2-positive GC treatment. However, unlike the success of various HER2-targeted agents in treating metastatic breast cancer [4, 5], only trastuzumab has been established to provide significant efficacy in HER2-positive GC patients, though trastuzumab resistance is increasingly common [6]. Thus, novel therapeutic strategies are urgently needed.

Clinical research has introduced antibody–drug conjugates (ADCs) as a novel approach to advancing treatment for trastuzumab-resistant HER2-positive advanced GC. ADCs efficiently conjugate monoclonal antibodies to small-molecule cytotoxic drugs through specific linkers for selective anti-tumor activity, providing a more precise and effective treatment for advanced GC [7]. The ADC Trastuzumab deruxtecan (T-Dxd), consists of a humanized monoclonal antibody targeting HER2, linked to the topoisomerase I inhibitor Dxd via a cleavable tetrapeptide linker [8]. While HER2-positive GCs have exhibited favorable clinical responses to T-Dxd for some patients, other patients experience limited efficacy, transient responses, and severe adverse effects. Thus, addressing the challenge of enhancing T-Dxd’s drug efficacy and minimizing adverse effects are pressing clinical concerns.

The therapeutic effectiveness of ADCs relies on their capacity to efficiently internalize and accumulate cytotoxic drugs within target cells. Consequently, enhancing drug endocytosis is a viable approach for improving both the efficacy and safety of ADCs [9]. However, the HER2 receptor presents significant resistance to internalization [10], and research to identify drugs that facilitate ADC drug endocytosis is limited. In a lung cancer cell line, simultaneous administration of the irreversible tyrosine kinase inhibitor (TKI) neratinib and the antibody-linked drug T-DM1 has been demonstrated to increase HER2 internalization and enhance anti-tumor activity, while the reversible HER2 inhibitor lapatinib resulted in reduced endocytosis [11], suggesting that the mechanism for reversible and irreversible HER2 inhibitors may differ. Nevertheless, there are no reported studies on the combination of HER2-TKIs and ADC in GC.

Herein, we provide a comprehensive examination of the roles of HER2-TKIs in improving the efficacy of T-Dxd in HER2-positive GC cells. We focused on their interactions, including their combined effects on HER2 protein levels, intracellular signaling, and therapeutic outcomes. Our goal was to reveal the mechanisms behind HER2-TKI and T-Dxd in treating HER2-positive GC, thus offering new insights for optimizing therapies and expanding clinical options.

## Materials and methods

### Cell lines and cell culture

The HER2-positive human GC cell lines NCI-N87 and SNU216 were kindly provided by the Beijing Institute of Genomics, Chinese Academy of Sciences. The cells were cultured in RPMI 1640 medium supplemented with 10% fetal bovine serum and 1% penicillin–streptomycin in a humidified atmosphere at 37 °C with 5% CO_2_.

### Plasmids and transfection

Plasmids encoding different domains of HER2 were kindly provided by Professor Yongliang Zhao from the Beijing Institute of Genomics, Chinese Academy of Sciences. Cell transfection was performed following the instructions of the DNA transfection reagent LipoMax (Sudgen, Nanjing, China).

### Western blot analysis

Protein extraction from cells and tissues was performed using RIPA buffer (Solarbio, Beijing, China). Protein concentrations were determined using a BCA protein assay kit (Thermo Fisher Scientific, Waltham, MA, USA). Equal protein amounts were separated by Sodium Dodecyl Sulfate–Polyacrylamide Gel Electrophoresis and transferred onto polyvinylidene fluoride membranes (Millipore, Massachusetts, MA, USA). The membranes were blocked for 1 h at room temperature with either 5% skimmed milk or 3% bovine serum albumin. Primary antibodies (Supplementary Table 1) were then applied, and the membranes were incubated overnight at 4 °C. After thorough washing, secondary antibodies were applied at room temperature for 1 h, and the protein bands were visualized using enhanced chemiluminescence.

### Co-immunoprecipitation

NCI-N87 and SNU216 cell lines were subjected to lysis using IP lysis buffer (Beyotime, Shanghai, China), followed by overnight incubation with either anti-HER2 or IgG antibodies at 4 °C. On the next day, 20 μL of Protein A/G PLUS-agarose beads were applied to each tube, with gentle rotation at 4 °C for 8 h. The beads were then subjected to a series of washes, and the resulting samples were analyzed via Western blotting to detect bands corresponding to ubiquitin, HER2, or HSP90.

### RNA extraction and qRT‑PCR

RNA was extracted from cells using TRIzol reagent (Invitrogen, Carlsbad, CA, USA). Subsequently, cDNA synthesis was carried out according to the manufacturer’s instructions using the PrimeScript™ RT reagent Kit (TaKaRa, Tokyo, Japan). Quantitative PCR (qPCR) was conducted with TB Green^®^ Premix Ex Taq™ II (TaKaRa, Tokyo, Japan). The data were normalized to the expression of the GAPDH internal control gene. The qRT-PCR primer sequences were as follows: HER2: forward, 5′-TGTGACTGCCTGTCCCTACAA-3′; and reverse, 5′-CCAGACCATAGCACACTCGG-3′. GAPDH: forward, 5′-GGAGCGAGATCCCTCCAAAAT-3′; and reverse, 5′-GGCTGTTGTCATACTTCTCATGG-3′.

### Hematoxylin–eosin (H&E) and Immunohistochemistry (IHC) staining

Tissue specimens were sectioned and stained with Hematoxylin and Eosin (H&E) or Immunohistochemistry (IHC). For the H&E staining, tissue sections were baked at 65 °C for 2 h, followed by automated staining. For IHC staining, paraffin-embedded tissue sections underwent dewaxing, hydration, antigen retrieval, and inhibition of endogenous peroxidase activity. Afterward, primary antibody was applied for 60 min, followed by secondary antibody for 15 min, after which 3,3′-Diaminobenzidine was used for color development. HER2 positivity was based on brownish yellow staining on the cell membrane and was quantified using ImageJ (National Institutes of Health, Bethesda, MD, USA).

### Immunofluorescence staining

Cells were seeded onto glass coverslips in 24-well plates (0.5 × 10^4^ cells per well) and cultivated for 24 h. Subsequently, the cells were fixed with 4% paraformaldehyde solution for 10 min. To enable permeabilization, 0.1% Triton X-100 solution was then applied for 10 min at room temperature. After thorough washing, the cells were incubated overnight at 4 °C with primary antibody. On the next day, the cells were rinsed in phosphate-buffered saline and then incubated with Alexa Fluor 488-labeled or Alexa Fluor 594-labeled secondary antibody at room temperature for 2 h. The cellular nuclei were stained with DAPI (Solarbio, Beijing, China) prior to mounting onto microscope slides and visualization by confocal microscopy (Nikon, Tokyo, Japan).

### pHrodo-ADC assay

To assess the impact of pyrotinib on T-Dxd endocytosis, the pHrodo™ Deep Red Antibody Labeling Kit (Invitrogen, Carlsbad, CA, USA) was used according to the manufacturer’s instructions. For our experimental setup, 0.5 × 10^4^ cells were seeded into 24-well plates with pre-placed coverslips. The cells were divided into two groups: the T-Dxd group and the T-Dxd combined with pyrotinib group. After overnight incubation, both groups received pHrodo-T-Dxd (1 μg/mL) at 4 h time points, while the combined group was supplemented with 0.1 μM pyrotinib 30 min subsequent to pHrodo-Dxd incubation. After treatment, the cells were fixed in 4% paraformaldehyde solution and visualized by laser confocal microscopy.

### In vitro cell growth assay

Cells were seeded in 96-well plates (8000 cells/well for NCI-N87, 3000 cells/well for SNU216). After overnight incubation, serial dilutions of pyrotinib or lapatinib were added. Cell viability was assessed after 24 h using CCK8 (ShareBio, Shanghai, China) according to the manufacturer’s instructions. To study synergy, the cells were divided into two groups: T-Dxd and T-Dxd combined with HER2-TKI. Based on the HER2-TKI results, doses selected with minimal proliferation impact for each cell type were combined with varying T-Dxd doses. After 24 h, the cell viability was measured using CCK8.

### In vivo xenograft studies

Animal experiments followed protocols approved by the Animal Research Committee of Bethune International Peace Hospital. Female BALB/c nude mice (5 weeks old) were obtained from the Vital River Laboratory Animal Technology Co., Ltd. (Beijing, China) and housed in a controlled, pathogen-free environment. Subcutaneous injections of 5 × 10^6^ NCI-N87 cells were administered into the right flanks, and treatments were initiated when the appropriate tumor volumes were reached. To mitigate gastrointestinal reactions linked to both HER-TKIs and T-Dxd, a lower intervention dose was selected. Xenografts were randomly assigned to treatment groups: T-Dxd (0.25 mg/kg, intravenously every 3 weeks) [12], pyrotinib (2 mg/kg, orally 5 days a week) [13], a combination of both, or vehicle control (sterilized water, orally 5 days a week). Daily observations were carried out to monitor potential toxicity. Tumor dimensions were measured biweekly using a digital caliper, and the tumor volume was calculated using the formula: tumor volume = (length × width^2^)/2. Subsequently, histological examinations, including H&E staining, IHC, and Western blotting assays were conducted on excised subcutaneous tumors.

### Statistical analysis

IBM SPSS Statistics Version 26.0 (IBM, New York, NY, USA) was used for data analysis and GraphPad Prism 8 (GraphPad Software, San Diego, CA, USA) for graph creation. Data were expressed as mean ± standard deviation. Two-group differences were evaluated by two-sample two-tailed Student’s *t*-test, while multiple-group comparisons were evaluated by one-way analysis of variance (ANOVA) with Dunnett’s post hoc test. A significance level was set at *α* = 0.05, with significance denoted as *P* < 0.05.

## Results

### Reversible and irreversible HER2-TKIs have contrasting effects on HER2 levels

**Fig. 1:**
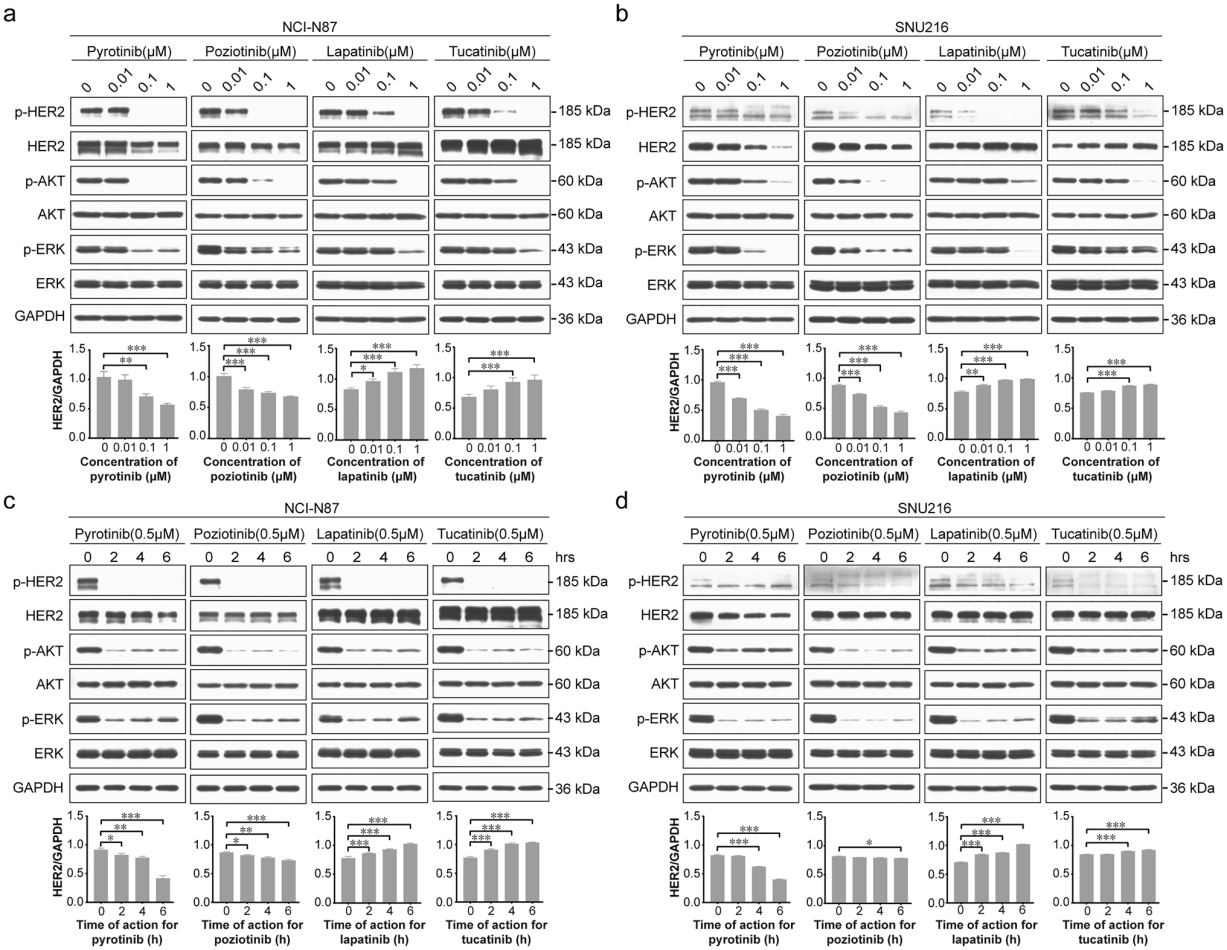
Modulation of HER2 expression and downstream signaling in HER2-positive gastric cancer (GC) cells by different HER2-TKIs. **a**, **b** NCI-N87 and SNU216 cells were treated with escalating concentrations of irreversible HER2-TKIs (pyrotinib or poziotinib) or reversible HER2-TKIs (lapatinib or tucatinib) for 24 h. **c**, **d** NCI-N87 and SNU216 cells were treated with irreversible HER2-TKIs (0.5 μM pyrotinib or poziotinib) or reversible HER2-TKIs (0.5 μM lapatinib or tucatinib) for specified time intervals. Western blot analysis was conducted to evaluate the impact on HER2 and p-HER2 expression and the activation of downstream signaling pathways. GAPDH served as the loading control. The graphs below the Western blots show the relative expression of HER2 protein. Significance levels: *P < 0.05, **P < 0.01, ***P < 0.001. Results are representative of 3 independent replicates

Given the central role of HER2-amplified GC in HER2-TKI treatment, we focused on the HER2-overexpressing GC cell lines NCI-N87 and SNU216. HER2 protein was overexpressed in both cell lines, but NCI-N87 cells exhibited significantly higher HER2 protein expression levels than SNU216 cells (Supplementary Fig. 1). We exposed the cells to varying concentrations and durations of reversible HER2-TKIs (lapatinib and tucatinib) and irreversible HER2-TKIs (pyrotinib and poziotinib). As expected, each of these treatments led to a concentration- and time-dependent suppression of p-HER2 levels, as well as decreases in downstream effectors, including p-AKT and p-ERK, underscoring the potency of the HER2-TKIs in inhibiting HER2 kinase activity (Fig. 1a–d). However, intriguingly, reversible HER2-TKIs and irreversible HER2-TKIs had distinctive impacts on HER2 total protein expression levels. The reversible HER2-TKIs lapatinib and tucatinib elicited a concentration- and time-dependent increase in HER2 protein abundance; while the irreversible HER2-TKIs pyrotinib and poziotinib induced a reduction in HER2 protein expression. These results suggest that reversible and irreversible HER2-TKIs display a fundamental difference in their mechanisms of action in HER2-positive GC.

### The irreversible HER2-TKI pyrotinib can enhance HER2 degradation by promoting HSP90 dissociation from HER2

**Fig. 2:**
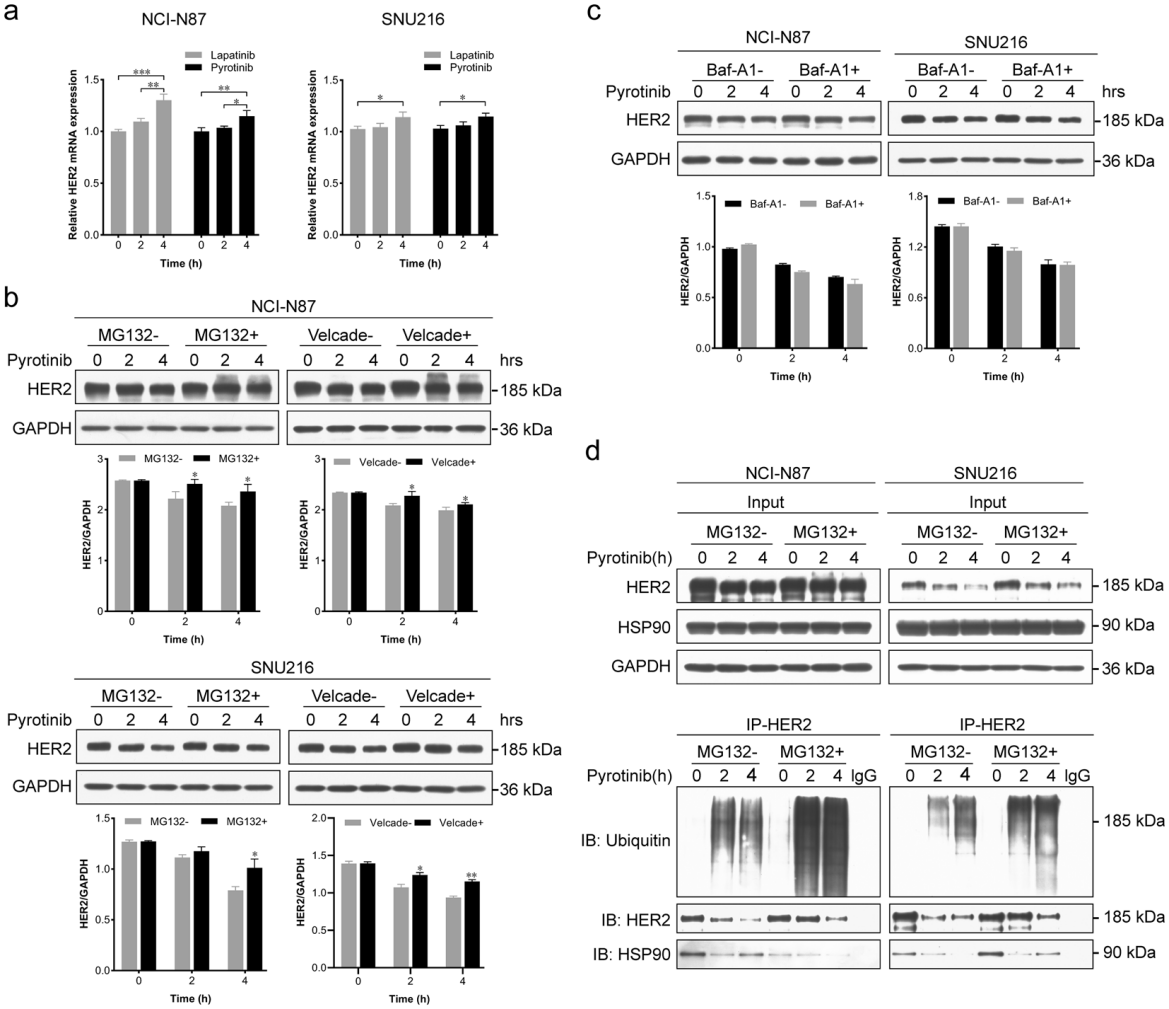
Pyrotinib enhances HER2 ubiquitination and subsequent degradation at the proteasome by inducing HSP90 dissociation from HER2. **a** HER2 mRNA levels were assessed in NCI-N87 and SNU216 cells treated with 0.5 μM pyrotinib or lapatinib by quantitative real-time polymerase chain reaction (qRT-PCR). **b**, **c** NCI-N87 and SNU216 cells were exposed to proteasome inhibitors MG132 (10 mM) or Velcade (0.5 μM) (**b**) or the lysosomal inhibitor Bafilomycin A1 (Baf-A1, 20 nM) (**c**) for 0.5 h, with DMSO as the control. Subsequently, pyrotinib (0.5 μM) was added for 2 or 4 h. Western blot analysis was employed to assess HER2 protein expression, with GAPDH serving as a loading control. The graphs below the Western blots show the relative expression of HER2 protein. **d** NCI-N87 and SNU216 cells were subjected to proteasome inhibition by MG132 (10 mM) for 0.5 h, with DMSO as the control. Subsequently, pyrotinib (0.5 μM) was added for 2 or 4 h. Immunoprecipitation of HER2 from cell lysates was performed, followed by immunoblotting with anti-ubiquitin, anti-HER2, and anti-HSP90 antibodies. GAPDH served as the loading control. Significance levels: *P < 0.05, **P < 0.01, ***P < 0.001. Results are representative of 3 independent replicates

To further elucidate the mechanisms underlying these changes, we conducted quantitative PCR experiments using the reversible HER2-TKI lapatinib and the irreversible HER2-TKI pyrotinib to assess HER2 mRNA levels following treatment. Surprisingly, a time-dependent increase in HER2 mRNA levels was observed upon exposure to both lapatinib and pyrotinib (Fig. 2a). This finding suggests that lapatinib-induced HER2 upregulation may be mediated transcriptionally, while pyrotinib-induced HER2 down-regulation may be mediated by post-transcriptionally.

The regulation of receptor stability, involving both ubiquitin–proteasome and lysosomal mechanisms, is an extensively documented post-transcriptional regulatory process [14, 15]. To determine whether the activity of pyrotinib in decreasing HER2 protein levels may be explained by effects on stability, we applied the proteasome inhibitors MG132 and Velcade and the lysosomal inhibitor Bafilomycin A1 (Baf-A1) to NCI-N87 and SNU216 cells 30 min prior to pyrotinib treatment. MG132 and Velcade promoted elevation of HER2 protein levels (Fig. 2b), while Baf-A1 had no impact on pyrotinib-induced HER2 degradation (Fig. 2c). These findings suggest that the irreversible HER2-TKI pyrotinib induces HER2 down-regulation via increased ubiquitin–proteasome degradation.

Molecular chaperone systems, among which HSP90 is included, play a recognized role in protecting client proteins, including HER2, from ubiquitin-mediated degradation [16]. This led us to postulate that pyrotinib might induce ubiquitination of HER2 by disrupting its association with HSP90. Consequently, we employed co-immunoprecipitation assays to assess the level of HER2 ubiquitin and its interaction with HSP90. The results demonstrate that after treatment with pyrotinib, HER2 ubiquitination was significantly enhanced, and the enhanced ubiquitination was accompanied by a notable reduction in the amount of HSP90 that co-immunoprecipitated with HER2, indicating that pyrotinib attenuates the interaction between HER2 and HSP90 (Fig. 2d). Conversely, lapatinib decreased HER2 ubiquitination (Supplementary Fig. 2), which may underlie a fundamental difference between reversible and irreversible HER2-TKIs.

To further elucidate the mechanism by which irreversible HER2 inhibitors prevent the binding of HSP90 to HER2 protein, we performed co-immunoprecipitation assay to determine the HER2 domain interacting with HSP90. The Flag-tagged truncated HER2 vectors were first transfected into HEK293 cells for 48 h. The result showed that only the intracellular tyrosine kinase domain exhibits interaction with HSP90 (Supplementary Fig. 3). Thus, irreversible HER2 inhibitor could hinder the HSP90 binding to HER2 through competitively targeting the tyrosine kinase domain.

### The irreversible HER2-TKI pyrotinib enhances HER2 internalization and facilitates T-Dxd endocytosis

**Fig. 3:**
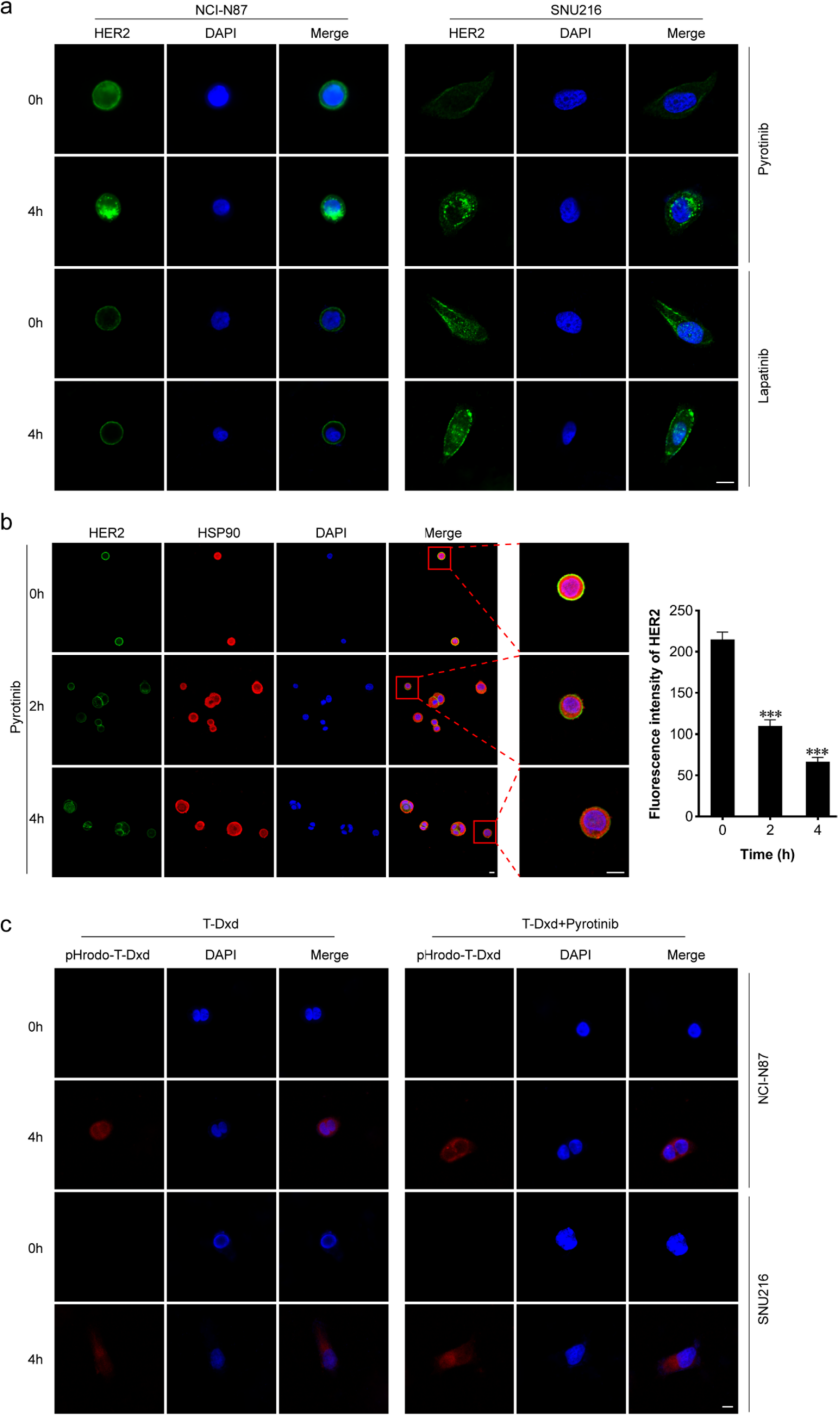
Pyrotinib enhances HER2 internalization and T-Dxd intracellular uptake. **a** NCI-N87 and SNU216 cells were treated with 0.5 μM pyrotinib or lapatinib for 0 or 4 h, and then the cells were prepared for immunofluorescence experiments using an anti-HER2 antibody (green). Nuclei were counterstained with DAPI (blue). **b** NCI-N87 cells were treated with 0.5 μM pyrotinib for 0, 2, or 4 h, and then the cells were prepared for immunofluorescence experiments using anti-HER2 antibody (green) and anti-HSP90 antibody (red). Nuclei were counterstained with DAPI (blue). The graphs on the right display the fluorescence intensity of the HER2 protein. **c** NCI-N87 and SNU216 cells were exposed to pHrodo-T-Dxd, marked by a red fluorescent pH-sensitive dye (pHrodo-T-Dxd, 1 μg/mL, which only emits light inside cells), or a combination of pHrodo-T-Dxd and pyrotinib (0.1 μM) for 0 or 4 h. Confocal microscopy captured images show intracellular red fluorescent dots as pHrodo-T-Dxd signals, with DAPI (blue) indicating nuclei. Scale bar = 10 μm. Significance levels: ***P < 0.001 vs 0 h. Results are representative of 3 independent replicates (colour figure online)

Receptors have the capacity to internalize from the cell’s outer membrane, leading to subsequent degradation via the ubiquitin–proteasome pathway [17]. To determine whether the induction of HER2 degradation by pyrotinib is mediated at the level of receptor endocytosis, we performed immunofluorescence assays. Our results in untreated NCI-N87 and SNU216 cells revealed predominant HER2 localization on the plasma membrane. Treatment with lapatinib led to increased HER2 accumulation on the plasma membrane, while treatment with pyrotinib resulted in a significant reduction in HER2 membrane distribution, which was accompanied by increased intracellular punctate structures (Fig. 3a). To elucidate the sequential interaction between HER2 and HSP90, NCI-N87 cells were treated with pyrotinib at various time intervals, followed by immunofluorescence staining for HER2 and HSP90. The findings revealed that in untreated NCI-N87 cells, HER2 and HSP90 are predominantly co-localized on the cell membrane. As the duration of pyrotinib treatment increased, the co-localization fluorescence intensity of HER2 and HSP90 on the cell membrane was progressively diminished (Fig. 3b). These observations suggest that the irreversible HER2-TKI pyrotinib may induce subcellular trafficking of HER2 by causing the dissociation of HSP90 from HER2.

To determine whether pyrotinib-induced HER2 internalization may facilitate T-Dxd endocytosis, we applied fluorescently labeled T-Dxd in the absence or presence of pyrotinib. In both NCI-N87 and SNU216 cells, red fluorescence could be detected after 4 h, indicative of T-Dxd endocytosis; however, the intracellular fluorescence signal of T-Dxd was more intense with pyrotinib co-treatment (Fig. 3c). These findings suggest that pyrotinib enhances T-Dxd intracellular uptake.

### Pyrotinib augments T-Dxd cytotoxicity in HER2-positive GC cells

**Fig. 4:**
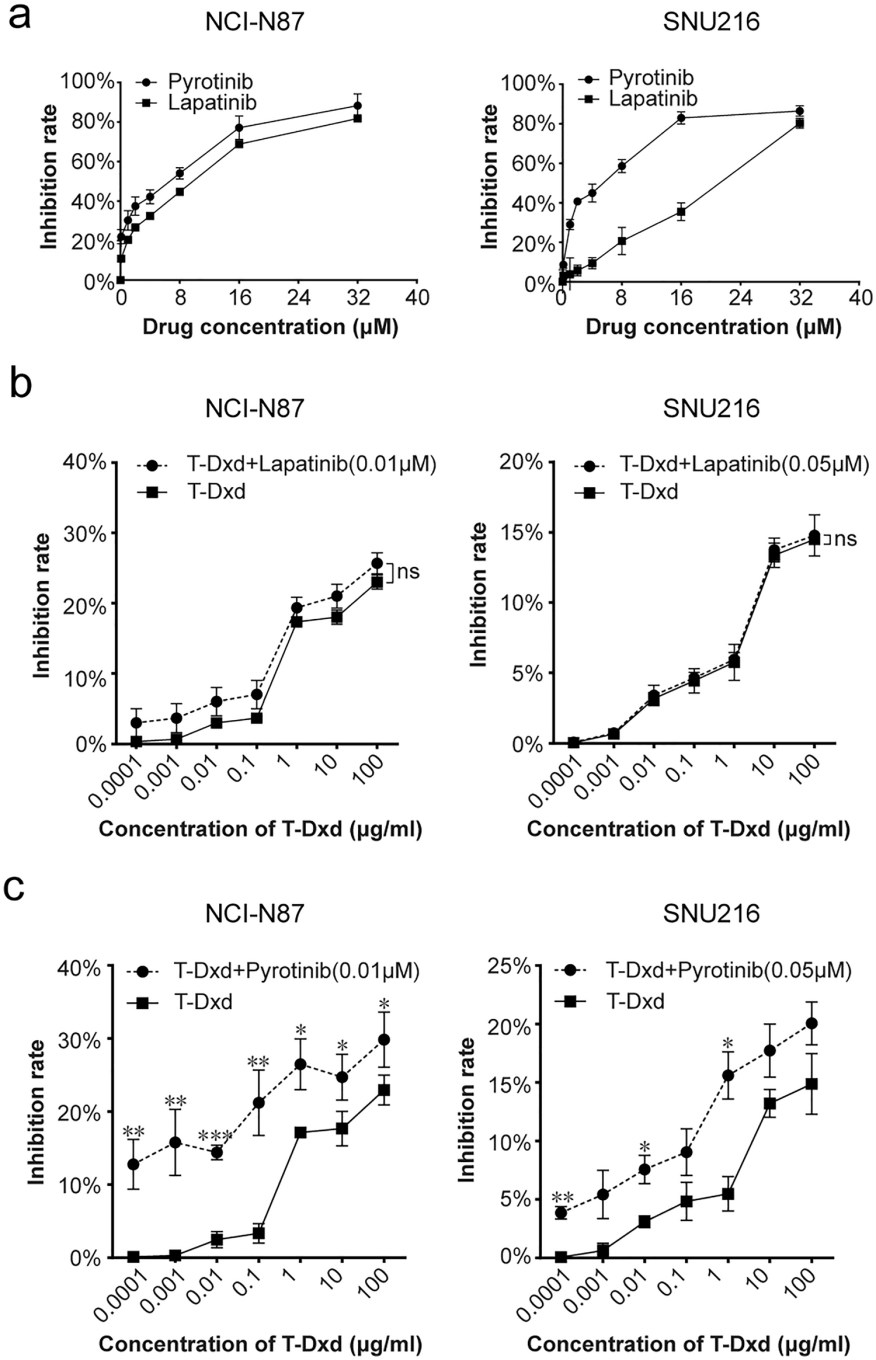
Pyrotinib amplifies T-Dxd’s inhibitory impact on HER2-positive GC cell proliferation. **a** NCI-N87 and SNU216 cells were treated with pyrotinib at defined concentrations, and the cell viability was assessed after 24 h. **b**, **c** NCI-N87 and SNU216 cells were exposed to different T-Dxd concentrations with or without a constant dosage of either lapatinib or pyrotinib. Cell viability was measured by CCK8 assay after 24 h of treatment. Significance levels: *P < 0.05, **P < 0.01, ***P < 0.001. *NS* not significant. Results represent means ± SD and are representative of 3 independent replicates

To evaluate the relevance of HER2-TKI-mediated induction of HER2 internalization and ubiquitination in terms of its therapeutic potential, we explored the cytotoxicity of T-Dxd alone or in combination with pyrotinib or lapatinib. First, we generated dose-curves of pyrotinib and lapatinib in NCI-N87 and SNU216 cells in the absence of T-Dxd to identify doses with mild effect on cell growth (Fig. 4a). The selected dosages (0.01 μM in NCI-N87 and 0.05 μM in SNU216) were subsequently paired with increasing T-Dxd doses. The outcomes revealed that the combination of lapatinib and T-Dxd was not significantly different from T-Dxd alone in terms of growth inhibition (Fig. 4b). In contrast, the pairing of pyrotinib with T-Dxd displayed a more potent growth inhibitory effect than that of T-Dxd monotherapy (Fig. 4c). These results suggest that non-toxic doses of pyrotinib can synergistically improve the cytotoxic effect of T-Dxd.

### Pyrotinib synergistically enhances T-Dxd tumor suppression in an NCI-N87 xenograft mouse model

**Fig. 5:**
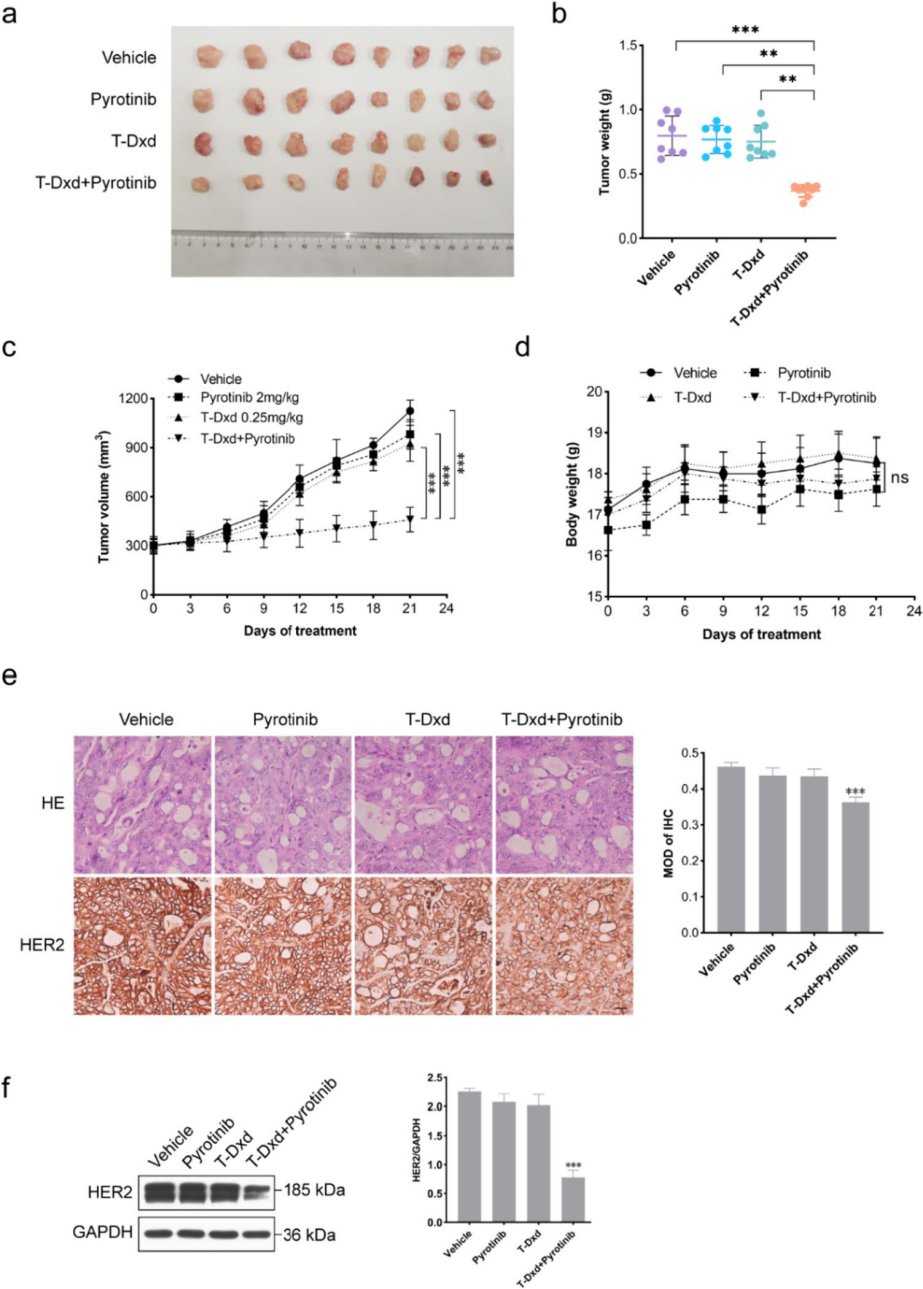
Enhanced in vivo antitumor effect of T-Dxd combined with pyrotinib. **a** Representative images of excised NCI-N87 xenografts after 21 days treatment (*n* = 8). **b** Eight tumors were weighed on day 21 for each treatment group. **c** Tumor volume changes were measured using digital calibers. **d** Body weight changes in mice after respective treatments. **e** Representative H&E and IHC-stained xenograft tumor tissues at × 400 magnification. Scale bars = 50 μm. **f** Quantification of HER2 protein expression in tumor tissues by western blotting, normalized to GAPDH. Significance levels: **P < 0.01, ***P < 0.001. *NS* not significant. Results are representative of 3 independent replicates

To validate our in vitro findings, we assessed the combined therapeutic effects of pyrotinib and T-Dxd using an NCI-N87 xenograft tumor model. When administered separately, pyrotinib and T-Dxd had limited effects on tumor growth. However, the combination of pyrotinib and T-Dxd exhibited an enhanced antitumor effect, surpassing the outcomes of monotherapy (Fig. 5a–c). Moreover, mice receiving the combination treatment compared to monotherapy experienced no significant change in body weight (Fig. 5d), indicating that the enhanced antitumor efficacy was observed without additional toxicity.

To further evaluate the in vivo synergistic effect of pyrotinib and T-Dxd in combination, we performed IHC staining of tumor tissues. Strong HER2 membrane staining (score 3 +) was observed in all groups. Notably, both the pyrotinib and T-Dxd single-agent groups displayed reduced HER2 staining compared to the control group, with a more pronounced reduction in the combination therapy group (Fig. 5e). Western blot analysis of the tumor tissues confirmed that HER2 protein expression was reduced in both the pyrotinib and T-Dxd single-agent groups compared to the control group, though the combination therapy group exhibited a significantly greater reduction in HER2 protein expression than in the other groups (Fig. 5f). These findings underscore the capacity of pyrotinib and T-Dxd to independently reduce HER2 protein expression, while their combined application synergistically enhances HER2 degradation.

## Discussion

In this study, we investigated the role of HER2-TKIs in regulating HER2-positive GC cells and their potential synergy with T-Dxd for targeted therapy of GC. Our findings reveal notable differences in the activities of reversible and irreversible HER2-TKIs and elucidate molecular mechanisms that may explain the effect of irreversible HER2-TKIs in decreasing HER2 protein levels and improving T-Dxd anti-tumor activity. These results offer valuable insights for advancing molecular targeted therapy for HER2-positive GC.

Notably, our results suggest that reversible and irreversible HER2-TKIs have distinct effects on the protein expression of HER2 that is regulated at the post-transcriptional level. While both reversible and irreversible HER2-TKIs inhibited HER2 phosphorylation and downstream signaling, the reversible HER2-TKIs lapatinib and tucatinib upregulated HER2 protein levels and the irreversible HER2-TKIs pyrotinib and poziotinib downregulated HER2 protein levels. Multiple studies have reported lapatinib’s ability to induce the accumulation of cell surface HER2 receptors [15, 18], which is consistent with our findings. Conversely, our results show that pyrotinib induces HER2 degradation through the ubiquitin–proteasome pathway, which is similar to the results of a study using the irreversible TKI CI-1033 for HER2-driven tumor cells [19]. In contrast, the irreversible HER2-TKI neratinib has been shown to induce HER2 degradation via the lysosomal pathway [20]. Therefore, the intracellular mechanism of HER2 degradation may vary for specific irreversible HER2-TKIs.

We observed that pyrotinib also increases HER2 ubiquitination by facilitating HSP90 dissociation from HER2, which is consistent with the established role of HSP90 in inhibiting HER2 degradation [21]. Conversely, lapatinib diminished HER2 ubiquitination, which may explain the opposing effects of reversible and irreversible HER-TKIs in modulating HER2 protein levels. Thus, differential effects on the HSP90-HER2 interaction may comprise a pivotal difference in the mechanisms of reversible versus irreversible inhibitors.

Our results also indicated that lapatinib promotes HER2 accumulation at the plasma membrane, whereas pyrotinib facilitates HER2 internalization. Because receptor internalization precedes degradation via the ubiquitin–proteasome pathway [17], these findings could partially explain the differential effects of reversible and irreversible inhibitors on HER2 protein stability. It is noteworthy that HER2 has been shown to exhibit a deficiency in endocytosis and resistance to down-regulation [22]; our results indicate that this deficiency is overcome by the irreversible TKI pyrotinib. Moreover, we demonstrated that pyrotinib significantly enhances the chemotherapeutic efficacy of T-Dxd, both in vitro and in vivo. Because the structural design of T-Dxd incorporates a HER2-targeting antibody [8], its increased efficacy when combined with pyrotinib is likely to be related to the enhanced HER2 endocytosis, leading to enhanced drug internalization. Significantly, in a preclinical patient-derived xenograft model of lung cancer, the combination of poziotinib with T-DM1 demonstrated superior activity [23]. However, in the latter study, the mechanism involved stabilizing HER2 at the membrane, which diverges from our research findings. Thus, there is a precedent for synergistic effects between irreversible HER2-TKIs and ADCs, though the mechanisms may differ depending on the specific drugs tested and the type of cancer.

In summary, our study elucidates intricate regulatory mechanisms governing HER2 expression that distinguish reversible and irreversible HER2-TKIs. Our results support the hypothesis that the enhanced efficacy of irreversible HER2-TKIs in combination therapy extends beyond inhibiting the HER2 downstream signaling pathway and may be related to enhanced HER2 endocytosis. The detailed mechanism of irreversible HER2-TKIs offers valuable insights for optimizing HER2-positive GC therapies. Additionally, our findings reveal the clinical potential of pyrotinib in enhancing ADC-mediated tumor cytotoxicity, making it a compelling combination therapy candidate for maximizing therapeutic efficacy while mitigating adverse drug reactions. These findings may provide insights for developing more effective treatment strategies for HER2-positive GC patients. While the study discusses the biological mechanisms leading to reduced HER2 protein levels, the associated molecular pathways or signaling networks may not be fully understood. Furthermore, our investigation affirms that irreversible HER2-TKI pyrotinib enhances the efficacy of T-Dxd. However, additional clinical studies are imperative to validate and optimize the optimal dosage of this two-drug combination.

## Conclusion

In this investigation, we observed a significant decrease in HER2 protein levels within HER2-positive GC cells upon treatment with irreversible HER2-TKI. This reduction was primarily driven by an increase in HER2 ubiquitination and subsequent proteasomal degradation, facilitated by the dissociation of HSP90 from HER2. Our analyses also revealed that the irreversible HER2-TKI pyrotinib actively promotes HER2 internalization. Importantly, combination of pyrotinib with the ADC drug T-Dxd substantially enhances the endocytosis of T-Dxd, leading to improved anti-tumor efficacy in both in vivo and in vitro models. The enhanced therapeutic benefits may facilitate lower drug doses and minimize the risk of adverse reactions. Further clinical investigations are needed to validate these preclinical findings and translate them into optimized therapeutic regimens for HER2-positive GC patients.

### Supplementary Information

Below is the link to the electronic supplementary material.

Supplementary file1 (DOCX 14 KB)

Supplementary file2 (DOCX 525 KB)
