# Supplementary material for: Elucidating molecular mechanisms and therapeutic synergy: irreversible HER2-TKI plus T-Dxd for enhanced anti-HER2 treatment of gastric cancer
Source: Gastric Cancer. 2024 Feb 22;27(3):495–505. doi: 10.1007/s10120-024-01478-6 (PMC11016512; doi:10.1007/s10120-024-01478-6)
Supplement: Supplementary file 1 — Supplementary file1 (DOCX 14 KB) [file 10120_2024_1478_MOESM1_ESM.docx]

**Supplementary Table 1: Primary antibodies for western blotting**

| Primary antibodies | Catalog Number | Dilution | Manufacturer | Producing areas |
| --- | --- | --- | --- | --- |
| HER2 | 2165 | 1:6000 | Cell Signaling Technology | Massachusetts, MA, USA |
| Phospho-HER2 | 2243 | 1:1000 | Cell Signaling Technology | Massachusetts, MA, USA |
| Phospho-AKT | 4060 | 1:2000 | Cell Signaling Technology | Massachusetts, MA, USA |
| Phospho-p44/42 MAPK (Erk1/2) | 9101 | 1:2000 | Cell Signaling Technology | Massachusetts, MA, USA |
| Ubiquitin | 43124 | 1:4000 | Cell Signaling Technology | Massachusetts, MA, USA |
| ERK | K23 | 1:2000 | Santa Cruz Biotechnology | Santa Cruz, CA, USA |
| AKT | 10176-2-AP | 1:2000 | Proteintech | Wuhan, China |
| HSP90 | 13171-1-AP | 1:3000 | Proteintech | Wuhan, China |
| GAPDH | 10494-1-AP | 1:10000 | Proteintech | Wuhan, China |
