# Supplementary material for: Elucidating molecular mechanisms and therapeutic synergy: irreversible HER2-TKI plus T-Dxd for enhanced anti-HER2 treatment of gastric cancer
Source: Gastric Cancer. 2024 Feb 22;27(3):495–505. doi: 10.1007/s10120-024-01478-6 (PMC11016512; doi:10.1007/s10120-024-01478-6)
Supplement: Supplementary file 2 — Supplementary file2 (DOCX 525 KB) [file 10120_2024_1478_MOESM2_ESM.docx]

**Supplementary Fig. 1** The protein expression of HER2 in NCI-N87 and SNU216 cells. Both NCI-N87 and SNU216 cells tested positive for HER2 expression by Western blot analysis, with NCI-N87 exhibiting a significantly higher expression level than SNU216. The data are presented as means ± standard deviations. *** *p* < 0.001.


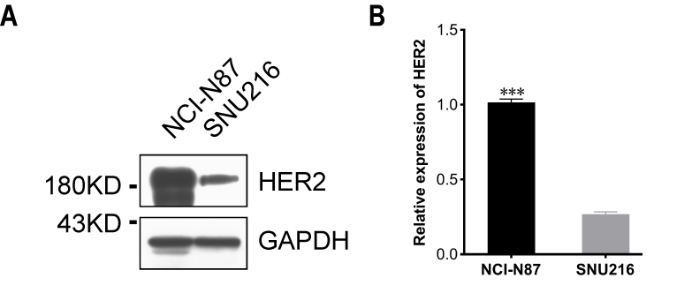


Supplementary Figure 1

**Supplementary Fig. 2** Effects of pyrotinib and lapatinib on HER2 Ubiquitination. Immunoprecipitation assays showed that pyrotinib treatment promotes HER2 ubiquitination, while lapatinib treatment decreases ubiquitination. Results are representative of 3 independent replicates.


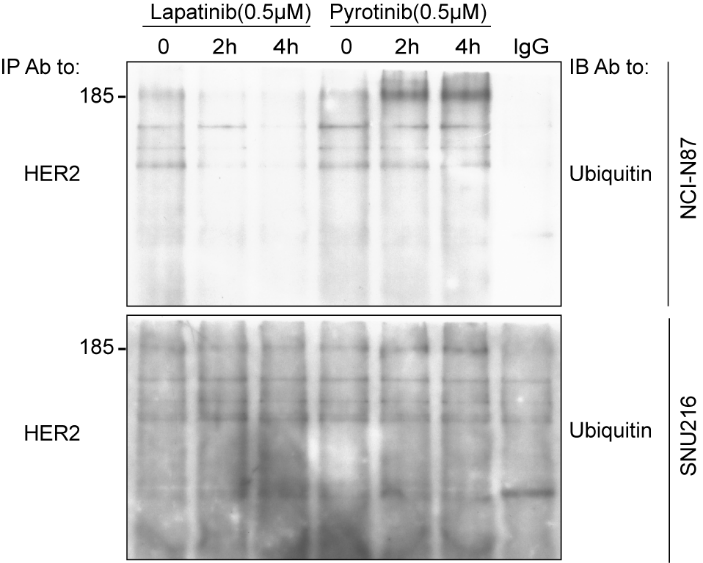


Supplementary Figure 2

**Supplementary Fig. 3** The interactions between HER2 domains with HSP90. Schematic diagram of HER2 truncated constructs for co-IP assays was shown in the upper panel. A list of Flag-tagged HER2 truncated fragments were constructed and overexpressed in HEK293 cells. Endogenous HSP90 was detected by Western blotting. RLD1: Receptor domain 1; FL: Furin-like cysteine rich region; RLD2: Receptor domain 2; GDRN Ⅳ: Growth factor receptor domain Ⅳ; ICD: Intracellular domain.


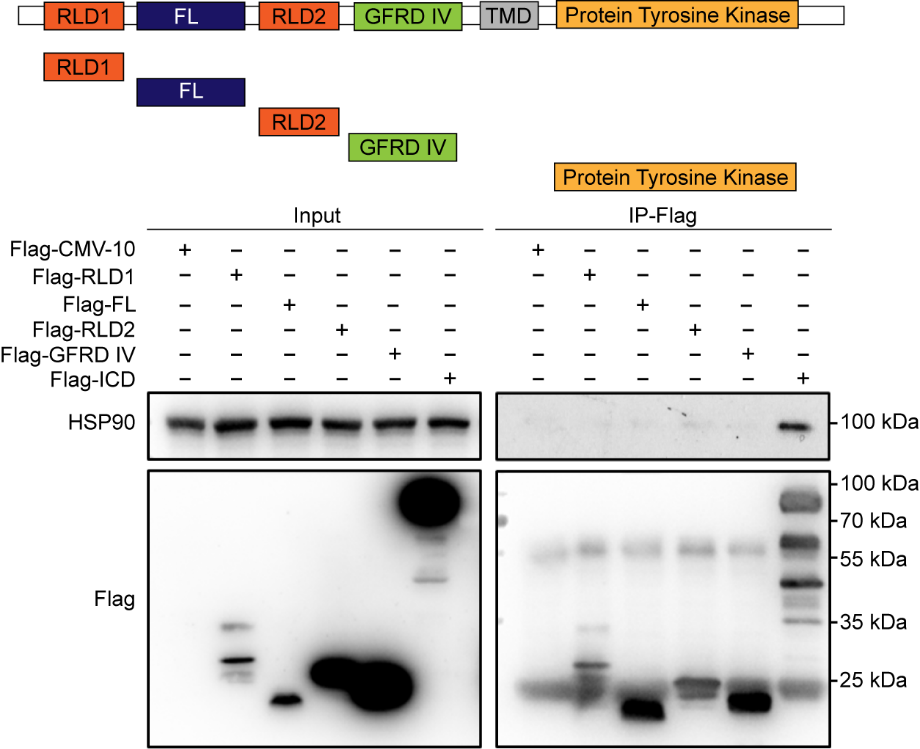


Supplementary Figure 3
